# Trends in Immediate Lymphatic Reconstruction

**DOI:** 10.7759/cureus.59194

**Published:** 2024-04-28

**Authors:** Shahnur Ahmed, Aladdin H Hassanein, Mary E Lester, Joshua Manghelli, Carla Fisher, Folosade Imeokparia, Kandice Ludwig, Betty Fan

**Affiliations:** 1 Surgery, Indiana University, Indianapolis, USA; 2 Plastic Surgery, Indiana University School of Medicine, Indianapolis, USA; 3 Plastic Surgery, Indiana University, Indianapolis, USA; 4 Surgical Oncology, Indiana University School of Medicine, Indianapolis, USA; 5 Breast Surgery, University of Chicago Medicine, Chicago, USA

**Keywords:** lympho-venous-anastomosis, alnd: - axillary lymph node dissection, axillary lymphadenectomy, breast lymphedema, breast cancer related lymphedema

## Abstract

Background and objective

Immediate lymphatic reconstruction (ILR) is emerging as a useful adjunct after axillary lymph node dissection (ALND), leading to a decrease in lymphedema rates from 30 to 3-13% in breast cancer patients. ILR requires coordination between two surgical specialties for oncologic ALND and microsurgical axillary lymphatic anastomosis. This study aimed to assess the trends in the frequency of ILR performed after ALND at our institution.

Methods

This study involved a retrospective review of breast cancer patients undergoing ALND with and without ILR at our institution (2017-2022). Data on patient demographics, tumor characteristics, and treatments received were gathered and analyzed.

Results

A total of 316 patients underwent ALND at our institution and 30.7% (97/316) of them received ILR. There was no significant difference in clinical breast cancer stages between patients who underwent ALND with or without ILR (p>0.05). Neoadjuvant chemotherapy was given to 51.1% (112/219) of patients with ALND only compared to 60.8% (59/97) of patients who underwent ALND with ILR (p=0.09). All patients received adjuvant radiation therapy. ILR was performed after ALND in 4.2% (2/47) in 2017, 25.8% (3/58) in 2018, 17.6% (12/68) in 2019, 35% (21/60) in 2020, 56.9% (41/72) in 2021, and 54.5% (6/11) in 2022. When comparing the first year of the ILR program with the last year of the study period, the odds ratio of receiving ILR after ALND was 1.8 (p=0.04).

Conclusions

The frequency of performing ILR after ALND in breast cancer patients at our institution witnessed a substantial increase during the study period. The implementation of an established ILR program at an institution can increase procedure uptake accompanied by continued growth in utilization.

## Introduction

Breast cancer-related lymphedema is an incurable disease process that results from disrupted lymphatics during axillary dissection. It is characterized by progressive limb swelling from adipose deposition, fluid stasis, and fibrosis [1]. Lymphedema rates in breast cancer patients undergoing axillary lymph node dissection (ALND) range from 30 to 50% [2-5]. Nonoperative strategies to manage lymphedema include limb elevation, static or pneumatic compression, and complex decongestive therapy [6]. Operative strategies to manage lymphedema include debulking procedures to reduce limb volume, vascularized lymph node transfer, lymphovenous bypass, and lymphovenous anastomosis [7]. 

Lymphoscintigraphy is a preoperative imaging modality to assess lymphatic vessel patency before microsurgical intervention in patients with longstanding lymphedema. Immediate lymphatic reconstruction (ILR) is a microsurgical technique that anastomoses disrupted axillary lymphatic vessels to nearby veins at the time of ALND and has been reported to reduce lymphedema rates from 30 to 4-12% [8,9]. Although ILR is now more frequently being studied and becoming more common, the current uptake rate of this procedure is unknown. ILR is only performed at centers with lymphatic reconstruction capabilities (e.g., microsurgery). In addition, even at centers that can offer the procedure, not all reconstructive surgeons may perform the operation. The purpose of this study is to evaluate the trend in the frequency of ILR uptake performed after ALND in breast cancer patients at our institution after the implementation of a designated program.

## Materials and methods

A single-center retrospective review of breast cancer patients treated at an academic health center and its satellite sites was conducted during a five-year period between 2017 and 2022. 

ILR was first established at our institution in 2017 and was a coordinated effort between breast and plastic surgery. ALND was performed using a standard technique for breast cancer treatment by a breast surgical oncologist as indicated. ILR was then performed immediately following ALND during the same operation by a plastic surgeon using microsurgical techniques. The use of axillary reverse mapping, number of channel anastomoses, and type of lymphatic channel-to-vein anastomosis (end-to-end versus end-to-side) were based on the surgeon's choice.

Breast cancer patients with stage I-III disease who underwent ALND with ILR and patients who underwent ALND without ILR were included in the analysis. Both patients who underwent neoadjuvant therapy or surgery upfront were included in the investigated cohort. Patients with incomplete records or who underwent ALND at an outside institution were excluded from this study. The frequency of ILR performed after ALND was assessed for each year. Patient demographic details, tumor characteristics, type of breast surgery, and perioperative treatments received were obtained.

Statistical analyses were performed using IBM SPSS Version 29 (IBM Corp., Armonk, NY). Two-tailed values of p<0.05 were considered statistically significant. Categorical variables were analyzed using chi-square tests. Continuous variables were compared with t-tests. Univariate analysis was used to calculate odds ratios. This study was approved by the Institutional Review Board (IRB) of the Indiana University School of Medicine.

## Results

During our study period, 316 breast cancer patients met the inclusion criteria and underwent ALND with or without ILR (Table 1). Of them, 69.3% (219/316) underwent ALND only compared to 30.7% (97/316) of patients who received ILR after ALND. Patients who underwent ILR + ALND were younger, with an average age of 51.4 ± 11.9 years, compared to those who underwent ALND with an average age of 55.3 ± 12.6 years (p=0.01). There were no statistically significant differences in BMI, rates of diabetes, or smoking status. The average BMI was 30.9 kg/m^2^ in patients with ALND only compared to 29.8 kg/m^2^ in the other group (p=0.211). Diabetes mellitus was present in 15% (33/219) of ALND-only patients compared to 10% (10/97) in patients who received ILR (p=0.255).

**Table 1:** Baseline characteristics of patients who underwent ALND only compared to patients who received ILR after ALND ALND: axillary lymph node dissection; ILR: immediate lymphatic reconstruction

Variables	ALND only (n=219)	ALND with ILR (n=97)	P-value
Age, years,	55.3 (12.6)	51.39 (11.9)	0.01
Body mass index, kg/m^2^, mean (SD)	30.9 (7.9)	29.8 (7.3)	0.211
Diabetes mellitus, n (%)	33 (15%)	10 (10%)	0.255
Smoker, n (%)	22 (10%)	12 (12%)	0.54
Breast cancer type, n (%)			
Ductal	196 (89.5%)	81 (83.9%)	0.12
Lobular	16 (7%)	9 (9.3%)	0.33
Mixed	7 (3%)	7 (7.2%)	0.21
Clinical stage, n (%)			
I	38 (17.4%)	22 (22.7%)	0.19
II	100 (45.6%)	43 (44%)	0.82
III	63 (28.8%)	26 (27%)	0.62
Neoadjuvant chemotherapy, n (%)	113 (51.6%)	59 (60.8%)	0.09

In our cohort, 87.6% (277/316) of patients had ductal type breast cancer, while 7.9% (25/316) had lobular type and 4.4% (14/316) had mixed type breast cancer. Of the patients who received ILR, 22.7% (22/97) were clinically stage I, 44.3% (43/97) were clinically stage II, and 26.8% (26/97) were clinically stage III. No significant difference was observed in breast cancer type or clinical cancer stage between patients who had ALND only or those with ILR after ALND (p>0.05). Neoadjuvant chemotherapy was given to 51.6% (113/219) of patients who had ALND only compared to 60.8% (59/97) of patients who received ILR (p=0.09). All patients who underwent ALND received adjuvant radiation.

**Figure 1:**
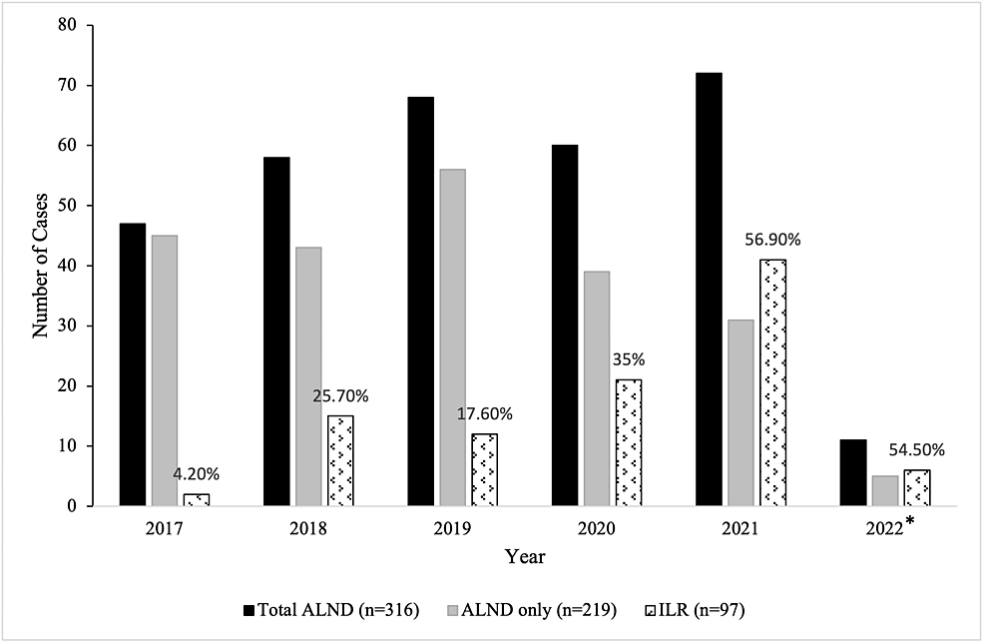
Comparison of ILR after ALND by year (rates of ILR are demonstrated in percentages) *Incomplete year ALND: axillary lymph node dissection; ILR: immediate lymphatic reconstruction

**Figure 2:**
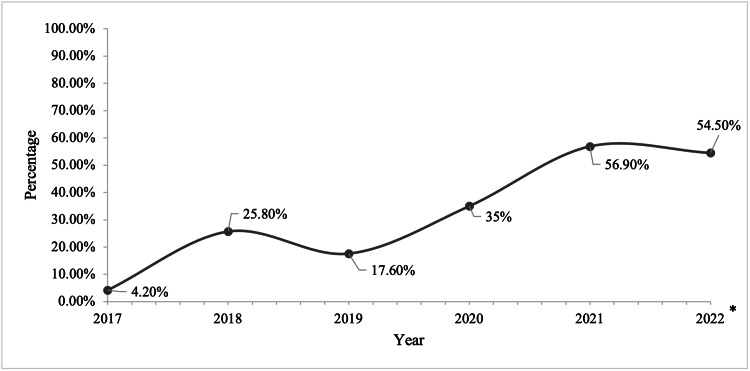
Yearly trend of ILR after ALND by percentage *Incomplete year ALND: axillary lymph node dissection; ILR: immediate lymphatic reconstruction

Rates of ILR after ALND performed in patients by year were as follows: 4.2% (2/47) in 2017, 25.8% (3/58) in 2018, 17.6% (12/68) in 2019, 35% (21/60) in 2020, 56.9% (41/72) in 2021, and 54.5% (6/11) in 2022 (Table 2, Figures 1-2). When comparing the first year of the study period with the last, the odds ratio of receiving ILR when an ALND was performed was 1.89 (p=0.04).

**Table 2:** Rate of ILR following ALND *Incomplete year ALND: axillary lymph node dissection; ILR: immediate lymphatic reconstruction

Year	Total ALND (n=316), n (%)	ALND only (n=219), n (%)	ILR (n=97), n (%)	% ILR by year
2017	47 (14.8%)	45 (20.5%)	2 (2.1%)	4.2% (2/47)
2018	58 (18.4%)	43 (19.6%)	15 (15.5%)	25.8% (15/58)
2019	68 (21.5%)	56 (25.6%)	12 (12.4%)	17.6% (12/68)
2020	60 (19%)	39 (17.8%)	21 (21.6%)	35% (21/60)
2021	72 (22.8%)	31 (31.9%)	41 (42.3%)	56.9% (41/72)
2022*	11* (3.48%)	5 (2.3%)	6 (61.9%)	54.5% (6/11)

## Discussion

Lymphedema is chronic limb swelling due to lymphatic dysfunction and affects up to 250 million people worldwide [10]. Skin thickening, interstitial fluid retention, and fibroadipose subcutaneous deposition from inflammation lead to progressive limb enlargement [11]. Morbidity associated with lymphedema includes recurrent arm cellulitis, pain, and impaired function of the affected extremity [12]. Axillary lymph node dissection performed in breast cancer patients can lead to lymphedema rates in 30-52% of cases [2]. Aside from compression and complete decongestive therapy, operative strategies such as debulking procedures, vascularized lymph node transfer, or lymphovenous bypass may reduce limb size and improve symptoms but do not cure lymphedema [13].

ILR is an emerging procedure to decrease lymphedema rates. Disrupted afferent lymphatics during axillary lymph node dissection are anastomosed to adjacent veins through microsurgical technique [3,5]. ILR decreases lymphedema occurrence to as low as 3.1% after ALND [2,3,4,9,14]. While ILR after ALND has shown promising results in improving lymphedema rates in several cohort studies, we sought to assess the trend of ILR at our institution. A formal ILR program was first established at our institution in 2017. All patients who are considered for ILR were seen preoperatively in the clinic by breast surgeons for oncologic surgical planning and plastic surgeons for ILR with or without breast reconstruction. In our study, there was a statistically significant difference in mean age between patients who underwent ALND only and those who had ILR after ALND (p=0.01). Older patients with potentially more comorbidities may not have opted for ILR as frequently so as not to extend the operation.

The presence of diabetes and smoking status were not significantly different between ALND-only patients and those receiving ILR, although these patient factors have been independently associated with surgical site infections after mastectomy [15]. ILR has been reported to add 32 to 90 minutes of additional operative time, which may further expose patients to potential complications associated with general anesthesia, potentially further worsened in high-risk patients [16]. The risks and benefits of this additional operative time should be considered at the time of preoperative consultation and ultimately be a shared decision between patient and physician.

Our results demonstrate an increased rate of ILR at the time of ALND for breast cancer patients at our institution, and its implementation has several benefits. Although our study did not look specifically into postoperative lymphedema rates, utilization of ILR has been evaluated by several cohort studies that report decreased lymphedema rates by more than 50% of its incidence (up to 30%), further highlighting the benefit of ILR implementation [2,3,5,8,9,14]. Johnson et al. analyzed the cost-effectiveness of ILR offered to patients undergoing ALND with or without postoperative radiation [17] and concluded that implementation of ILR in these procedures was more cost-effective than going without ILR. One limitation of ILR being offered more universally at centers that have technical capabilities is a lack of a specific common procedural terminology (CPT) code for ILR. Insurance reimbursements can be complicated or the procedure may even be uncovered.

Our study has a few limitations, primarily its retrospective design. While we did not assess the operative time in our study, the additional time associated with ILR procedures and disruption in operating schedules can be a hurdle for institutions developing a new ILR program, particularly if performed with intraoperative frozen sections [16]. However, as our study reflects, the implementation of a new ILR program can result in effective uptake of this procedure with continued growth in utilization. Furthermore, details regarding patients who did not receive ILR were not collected and should be further analyzed to evaluate potential areas of improvement.

## Conclusions

ILR has shown consistent promise in decreasing rates of lymphedema after ALND for breast cancer. The frequency of ILR after ALND in breast cancer patients witnessed a significant increase during the study period. The implementation of an established ILR program at an institution can increase procedure uptake accompanied by continued growth in utilization.
